# What is a “high” prevalence of obesity? Two rapid reviews and a proposed set of thresholds for classifying prevalence levels

**DOI:** 10.1111/obr.13363

**Published:** 2021-09-28

**Authors:** Tim Lobstein, Jo Jewell

**Affiliations:** ^1^ Policy Section World Obesity Federation London UK; ^2^ The Boden Group University of Sydney Sydney New South Wales Australia; ^3^ Nutrition Section UNICEF New York New York USA

**Keywords:** advocacy, intervention, prevalence threshold, risk assessment

## Abstract

Categories such as “low” and “high” have been used for several decades to describe the prevalence of stunting and wasting in populations of children aged under 5 years. They provide support for public health risk assessment and policy‐making, including alerting health departments and aid agencies to national trends and local needs. In the light of the need for monitoring progress to meet globally agreed targets for overweight and obesity, the classification of their prevalence will be a valuable to aid in policy development, to target resources, and to promote public health interventions. This paper reviews the current use of categories to describe obesity prevalence in policy, advocacy, and research literature. Where prevalence categories have been formally proposed, this paper compares their application on large‐scale datasets. The paper then develops a set of recommended threshold values to classify prevalence levels for overweight and obesity among children under age 5 years, children aged 5–19 years, and adults.

## 1 INTRODUCTION

Excess bodyweight in childhood affects more than 380 million children and adolescents,^1^ of whom the great majority, 82%, live in low‐ and middle‐income countries (LMICs). In these countries, child overweight has risen rapidly: In just 6 years from 2010 to 2016, the estimated prevalence of overweight increased by 33% in Sub‐Saharan Africa, 43% in Western Pacific, and 48% in South‐East Asia.^2^

In 2013, members of the World Health Assembly agreed to work towards a target, by 2025, of a 25% reduction in mortality for NCDs and no increase in the prevalence of adult obesity or diabetes above 2010 levels.^3^ In 2015, the UN General Assembly adopted the 2030 Agenda for Sustainable Development^4^ with a suite of goals including, by 2030, reducing by one third of premature mortality from NCDs and ending “all forms of malnutrition.”

Among the many policy developments, in 2020, UNICEF and the World Health Organization (WHO) started piloting tools for strengthening country‐level responses, assessing children's nutritional status and the risk factors for overweight and obesity.^5^ The risk assessment tools include proposed classifications for the prevalence of obesity and overweight in terms of whether “low” or “high.” The present paper describes the process undertaken to define these classifications, reviewing the use of classification schemes and the rationale for the thresholds proposed.

### 1.1 Current use of obesity prevalence categories

In order to understand better the descriptions of obesity and overweight prevalence levels used in the literature, a rapid review was undertaken of published anthropometric surveys to assess how the descriptor “high” may be used to describe obesity prevalence.

## 2 METHODS

### 2.1 Prevalence surveys

The National Library of Science database (PubMed) was searched in early October 2020 for published papers using the word “high” associated with a report of obesity prevalence, published in the previous 25 years (September 1995 to September 2020). Of more than 30,000 titles returned, the first 200 “best match” were examined. Papers were excluded if they used comparators such as “higher than” and “not as high as” or did not report surveys of children's or adults' adiposity prevalence. Full text papers were examined by one author.

### 2.2 Public health strategies

In a second survey, public health obesity strategy papers were examined for references to threshold values for obesity prevalence levels. A literature search was undertaken using Google and Google Scholar for documents describing obesity prevalence and sourced from government departments, intergovernmental agencies, and obesity professional societies (members of the World Obesity Federation). The first 200 returns were examined. Additional documents were examined based on the references cited in the retrieved documents and following contacts with authors (B Popkin and M Shekar). Papers were included if they defined the criteria used for classifying prevalence levels in population groups.

### 2.3 Development of an expanded set of prevalence thresholds

In the analyses described below, the calculations of correlation coefficients use Pearson's product moment calculations, using the Microsoft Excel® “CORREL” function, and the calculation of regressions using the Microsoft Excel® scatterplot facility with the associated regression equation (linear regression unless otherwise stated). Scatterplot graphs are shown in the supporting information available online.

## 3 RESULTS

### 3.1 Prevalence surveys

Of the 200 “best match” records returned, 31 were excluded for using the term relatively (e.g., not as high, higher, and highest), 64 were excluded for using the term for comorbid conditions (e.g., high blood pressure and high risk of obstetric problems), and 14 were excluded for using the term to describe subjects or locations (e.g., high school, high income, and high mountain). The remaining 91 records were inspected by the first author and 50 of these were deliberatively selected to demonstrate the range of prevalence levels and range of population samples where the term “high” was being used to describe obesity prevalence. The results are shown in Table 1, where it can be seen that the term is applied to a wide variety of prevalence levels, and based on several different criteria for defining overweight or obesity. The lowest levels of prevalence described as “high” were below 10% for adults and below 5% for children and adolescents. None of the papers referred to a published definition of “high,” and the use of the term “high” appeared to be based on the authors' own judgments. These judgments may have been based on authors' expectations: For example, in surveys of adults in LMICs, the word “high” was used for adult prevalence at levels as low as 6.8% in Nigeria and 5.2% in Malaysia. For children, “high” was used to describe levels of obesity prevalence below 10% in both low‐ and high‐income countries, for example, 4.6% in India, 5% in Norway, 5.4% in Switzerland, and 3.9%–5.1% in Sweden, and the phrase “too high” was used to describe prevalence levels of 3.1%–4.4% among children in France.

**TABLE 1:** Examples of use of the word “high” to describe obesity prevalence 1995–2020, listed by author alphabetically

Source	Country	Age group and adiposity definition	Descriptor	Prevalence
Ajlouni et al. (2020)^6^	Jordan	Adults: obesity WC ≥94 for men and ≥80 for women	Alarmingly high	Men 60.4%; women 75.6%
Al Junaibi et al. (2013)^7^	United Arab Emirates	Children 6–19 years: obesity BMI > 95th centile (CDC)	High	18.9%
Anwar et al. (2010)^8^	India	Children 11–14 years: obesity BMI > 97th centile (WHO)	High	11.9%
Arroyo et al. (2000)^9^	Mexico	Adults: obesity BMI > 30	High	21%
Bautista‐Castaño et al. (2011)^10^	Spain (Canaries)	Adult pregnant women: obesity BMI > 30	High	17.1%
Berg et al. (2001)^11^	Sweden	Young people 12–18 years: obesity BMI > 98th centile (UK90)	High	Boys 7.3%–8.9%, girls 3.9%–5.1%
Castetbon (2015)^12^	France	Children 5–15 years: obesity IOTF ≡ adult BMI > 30	Too high	3.1%–4.4%
Charo et al. (2014)^13^	USA	Children 2–19 years: obesity BMI > 95th centile (CDC); adults BMI > 30	High	Children 16.9%; adults 34.9%
Chigbu et al. (2018)^14^	Nigeria	Adults: obesity BMI > 30	High	6.8%
Colmegna et al. (2016)^15^	Canada	Adults with rheumatoid arthritis: obesity BMI > 30	High	34%
Coronado Vázquez et al. (2012)^16^	Spain	Children 6–14 years: obesity IOTF ≡ adult BMI > 30; obesity BMI > 95th centile (CDC)	Very High	IOTF: 11.6% CDC: 16.5%
De Pablos‐Velasco et al. (2002)^17^	Spain (Canaries)	Adults >30 years: obesity BMI > 30	Extremely high	Men 36.5%; women 23.6%
del Río‐Navarro et al. (2000)^18^	Mexico	Children 10–17 years: obesity IOTF ≡ adult BMI > 30; obesity BMI > 95th centile (CDC)	High	IOTF: Boys 11.6%, girls 9.5% CDC: Boys 7.7%, girls 6.9%
Farsi and Elkhodary (2017)^19^	Saudi Arabia	Adolescent boys mean age 16.5 years: obesity BMI > 95th centile (Saudi tables)	High	50.5%
Fernald et al. (2004)^20^	Mexico	Adults: obesity BMI > 30	High	Men 13.6%; women 22.2%
Freitas et al. (2013)^21^	Brazil	Adults: Abdominal obesity WC > 94 for men and WC > 80 for women	High	44.8%
Galfo et al. (2016)^22^	Italy	Adolescents 15–16 years: obesity IOTF ≡ adult BMI > 30	High	7.9%
Ganie et al. (2017)^23^	India	Children 6–18 years: obesity BMI > 95th centile (CDC)	High	4.6%
Gezawa et al. (2013)^24^	Nigeria	Adults: obesity BMI > 30	High	17.1%
Goday‐Arnó et al. (2013)^25^	Spain	Adults: obesity BMI 30–39.9	High	14.5% (men 17.0%, women 7.7%)
Gofin et al. (1996)^26^	Israel	Adults: obesity BMI > 30	High	Men 16%; women 33%
Gopalakrishnan et al. (2012)^27^	Malaysia	Adult students 19–25 years: obesity BMI > 30	High	5.2%
Grammatikopoulou et al. (2013)^28^	Greece	Adult pregnant women: obesity BMI > 30	High	25.6%
Grol et al. (1997)^29^	Curacao	Adults: obesity BMI > 30	Alarmingly high	Men 19%; women 36%
Grujić et al. (2009)^30^	Serbia	Adults: obesity BMI > 30	High	Men 20.2%; women 23.1%
Hedley et al. (2004)^31^	USA	Children 6–19 years: overweight BMI > 95th centile (CDC); adults: obesity BMI > 30	High	Children 16.0%; adults 30.4%
Herlevic et al. (2015)^32^	USA	Adult women: obesity BMI > 30	High	55%
Ichinohe et al. (2005)^33^	Jamaica	Adult women: obesity BMI > 30	Very high	23.9%
Isasi et al. (2011)^34^	USA	Children 11–18 years: obesity BMI > 95th centile (CDC)	High	22.5%
Jiang et al. (2014)^35^	China	Children 8–15 years: obesity IOTF ≡ adult BMI > 27 [*and ≡ adult BMI > 30*]	High	Boys 19.8%; girls 8.4% [*boys 7.9%; girls 2.7%*]
Johnson‐Down et al. (1997)^36^	Canada	Children 9–12 years: obesity BMI > 95th centile (NHANES II)	High	19.4%
Kokkvoll et al. (2012)^37^	Norway	Children 6 years: obesity IOTF ≡ adult BMI > 30	High	5%
Ledergerber and Steffen (2011)^38^	Switzerland	Children 5–16 years: IOTF ≡ adult BMI > 30	High	5.4%
Malik and Bakir (2007)^39^	United Arab Emirates	Children 5–17 years: overweight IOTF ≡ adult BMI > 25	High	21.5%
Mkuu et al. (2018)^40^	Kenya	Adult women: obesity BMI > 30	High	9.1%
Nafiu et al. (2007)^41^	USA	Children 2–18 years: obesity BMI > 95th centile (CDC 2000)	High	17.2%
Nathell et al. (2002)^42^	Sweden	Adults with asthma: obesity BMI > 30	High	20.7%
Nguyen et al. (2013)^43^	Vietnam	Children 11–14 years: overweight IOTF ≡ adult BMI > 25	High	Boys 22.0%; girls 13.3%
O'Neill et al. (2007)^44^	Ireland	Children 5–12 years: overweight BMI > 95th centile (CDC 2000); obesity BMI > 98th centile (UK90); obesity IOTF ≡ adult BMI > 30	High	CDC: 10.7% UK90: 11.2% IOTF: 7.2%
Padez et al. (2004)^45^	Portugal	Children 7–9 years: obesity IOTF ≡ adult BMI > 30	High	11.3%
Papadimitriou et al. (2006)^46^	Greece	Children 6–11 years: obesity IOTF ≡ adult BMI > 30	High	Boys 12.3%; girls 9.9%
Papadimitriou et al. (2008)^47^	Greece	Adult men 19–26 years: obesity BMI > 30	Alarmingly high	10.4%
Pedrosa et al. (2011)^48^	Portugal	Children 7–9 years: obesity IOTF ≡ adult BMI > 30; and BMI > 98th centile (CDC)	High	IOTF: 8.1% CDC: 14.0%
Preston et al. (2015)^49^	Peru	Children 7–8 years: obesity BMI > 2sd (WHO)	High	8.6%
Ruangkanchanasetr et al. (2014)^50^	Thailand	Adults: “obesity” BMI > 25	High	39.7%
Salimi et al. (2019)^51^	Iran	Adults: obesity BMI > 30	High	13%
Somasundaram et al. (2019)^52^	Sri Lanka	Adults: obesity BMI > 30	High	15.8%
Stewart et al. (2009)^53^	Scotland UK	Children mean age 13.3 years: obesity BMI > 95th centile (UK90)	High	36%
Vrazić et al. (2012)^54^	Croatia	Adults: obesity BMI > 30	High	28.6%
Yumuk et al. (2005)^55^	Turkey	Adults: obesity BMI > 30	High	23.7%

Abbreviations: BMI, body mass index (in kilograms per meter squared); CDC 2000, US Centers for Disease Control and Prevention 2000 growth reference tables; IOTF, International Obesity Task Force growth reference tables; NHANES II, US National Health and Nutrition Examination Survey 1990 growth reference tables; UK90, United Kingdom 1990 growth reference tables; WC, waist circumference (in centimeters); WHO, World Health Organization 2007 growth reference tables.

### 3.2 Public health strategies

Of 200 records returned by the searches, 130 records were excluded as not relating to obesity or related conditions (58 of the records), as social media links (24 records), advertising (43 records), and foreign language (5 records). Of the remaining 70 records, documents were downloaded and examined in detail. Of these, a further nine were found to be duplicates held on different sites, leaving 46 governmental and intergovernmental documents and 15 professional society documents included for examination. Documents in which the classification of prevalence levels for overweight or obesity were formally defined were examined and the source reference extracted: These source references were associated with four organizations, namely, the WHO together with UNICEF (cited 53 times),^56^, ^57^ the World Bank^58^ linked to a paper by Popkin et al.^59^ (together cited 6 times), and the World Obesity Federation (cited 2 times).^60^, ^61^ The details of the prevalence classifications are shown in Table 2. All papers use the same definitions for overweight and obesity, based on the WHO criteria.^62^ A fifth source, *The 2020 Global Nutrition Report*,^63^ stated in footnotes that the authors used a definition for “high” prevalence of overweight (including obesity) among adult women as being greater than 35%, but the document provides no source. This threshold is close to the definition used by the World Bank Group, shown in Table 2.

**TABLE 2:** Prevalence classifications and thresholds proposed by four sources

Children under 5 years				
WHO standards: Weight for height overweight (>2sd)				
	World Health Organization/UNICEF^57^	World Bank Group^58^	Popkin et al.^59^	World Obesity Federation^60^
Very low	<2.5%			
Low	2.5% to <5%	<10%		<5% (green)
Medium	5% to <10%	10% to <15%	>20%	5% to 15% (amber)
High	10% to <15%	15% to <20%	>30%	>15% (red)
Very high	≥15%	≥20%	>40%	

Older children and adolescents 5–19 years				
WHO reference: Overweight (BMI > 1sd), obesity (BMI > 2sd)				
		World Bank Group^58^ overweight	Popkin et al.^59^ overweight	World Obesity Federation^60^ obesity
Very low				
Low		<10%		<5% (green)
Medium		10% to <15%	>20%	5% to 15% (amber)
High		15% to <20%	>30%	>15% (red)
Very high		≥20%	>40%	

Adults				
Overweight (BMI > 25), obesity (BMI > 30)				
		World Bank Group^58^ overweight	Popkin et al.^59^ overweight	World Obesity Federation^61^ obesity
Very low				
Low		<20%		<5% (green)
Medium		20% to <30%	>20%	5% to 15% (amber)
High		30% to <40%	>30%	>15% (red)
Very high		≥40%	>40%	

Abbreviations: BMI, body mass index; WHO, World Health Organization.

### 3.3 Developing a uniform set of criteria

#### 3.3.1 Criteria for overweight prevalence among children under age 5 years

As noted, the purposes of classifying prevalence levels are to assist public health risk assessment, policy development and policy monitoring and improve public communication. The question arises as to what validation measures can be used to justify a preference for one classification scheme over another. The paper by de Onis et al.^56^ for children under 5 years uses prevalence categories defined and widely accepted for wasting and applies them to overweight, on the basis that both measures are at either end of the same distribution continuum (i.e., weight for length or height). De Onis et al.^56^ also justify their criteria by showing that the range of values for national prevalence found in 128 countries reporting up to 2017 are similar for wasting (range: 0.1% to 22.4%) as they are for overweight (range: 0.1% to 26.5%). This might change according to the success or failure of policies to tackle malnutrition, but the updated figures for 2021, expanded to 155 countries, show prevalence ranging from 0.0% to 22.7% for wasting and 1.3% to 25.4% for overweight,^64^ indicating that a similar equality of distribution has been maintained in the most recent years and applies across a greater number of countries. Within each classification, the proportion of countries has also remained consistent in the most recent years, as shown in Table 3.

**TABLE 3:** Distribution of countries' prevalence of overweight in children under age 5 years, for 2017 and 2021 classified according to de Onis et al. 2019^56^

		2017		2021	
		De Onis et al.^56^		UNICEF/WHO/World Bank^64^	
WHO classification	Prevalence	Number of countries		Number of countries	
Very low	<2.5%	18	14%	17	11%
Low	2.5% to <5%	33	26%	40	26%
Medium	5% to <10%	50	39%	68	44%
High	10% to <15%	18	14%	23	15%
Very high	≥15%	9	7%	7	5
		*128*	*100%*	*155*	*100%*

Abbreviation: WHO, World Health Organization.

It can also be argued that the definition of a “very low” prevalence of overweight at a threshold of 2.5% of the population has some external validation, given that a population of healthy children used by WHO to represent optimum growth defines overweight at a threshold of weight‐for‐height *Z* score above +2.0, equivalent to 2.3% of the population.^65^

Given these arguments, the de Onis et al.^56^ classification scheme was adopted for the joint WHO–UNICEF 2019 publication *Recommendations for data collection, analysis and reporting on anthropometric indicators in children under 5 years old*.^57^ As Table 2 shows, the WHO–UNICEF classification scheme is not identical to that suggested by either the World Bank or the paper by Popkin et al. Table 4 compares the distribution of prevalence classifications using the WHO–UNICEF criteria and the World Bank Group criteria, for the 155 countries reported in the 2021 *Joint Malnutrition Estimates*.^64^

**TABLE 4:** Children under age 5 years: Comparison of number of countries classified according to World Bank Group and WHO/UNICEF classifications of overweight prevalence for 155 countries

		World Bank classification			
		Low	Medium	High	Very high
WHO classification	*n*	*125*	*23*	*6*	*1*
Very low	*17*	17			
Low	*40*	40			
Medium	*68*	68			
High	*23*		23		
Very high	*7*			6	1
	*155*				

Under the World Bank categorization, 81% of countries are classified with a “low” prevalence of overweight, and less than 5% of countries with a “high” or “very high” prevalence, and this is similar to the Popkin et al. scheme which would classify 99% of countries as having less than a “medium” prevalence of overweight. This suggests that the discriminatory power of either of these two classification schemes may be relatively weak for distinguishing countries, and from a public health perspective, they may lead to many countries assuming that obesity prevention in younger children is unnecessary. For that reason, the present paper recommends using the WHO–UNICEF proposals for children under age 5 years (see the recommendations summarized in Table 7).

#### 3.3.2 Criteria for obesity prevalence among children aged 5–19 years

The classification of overweight prevalence among children under 5 years can be used as a basis for developing threshold criteria for categorizing obesity in older children. The WHO defines obesity in older children and adolescents (age 5–19 years) as having a body mass index (BMI) *Z* score above +2.0, compared with a reference population. In the case of BMI distribution, the same logic can be followed, with <2.5% being the threshold for “very low” as again there would be an expected 2.3% of children to have a BMI above a *Z* score of +2.0 in a healthy population for this age range. Applying the same classification scheme used for children under age 5 years to children aged 5–19 years, the distribution of prevalence levels for 191 countries provided in the WHO Global Health Observatory^66^ shows 14% of countries to have “very low” obesity prevalence and 12% “very high” obesity prevalence (Table S1). Although there are many reasons why overweight in under‐5s may not translate into obesity in children 5–19 years, a linear correlation between the two prevalence estimates for 152 countries (the countries where both estimates are available) is statistically significant (*r* = 0.42, *p* < 0.001).

#### 3.3.3 Criteria for obesity prevalence among adults

The next step moves from child to adult prevalence. A very high correlation (*r* = 0.91, *p* < 0.001) is found between child and adolescent (ages 5–19 years) obesity prevalence and age‐adjusted adult obesity prevalence, across 192 countries in the WHO Global Health Observatory's 2016 estimates.^67^ The regression equation (*y* = 0.51*x* − 1.20) indicates that adult obesity prevalence is found at approximately twice the levels found in children and adolescents (see Figure S1). On this basis, it is reasonable to suggest categories for adults based on double the prevalence thresholds for children aged 5–19 years.

However, based on the regression equation, the equivalent figure for 10% prevalence in children is around 23% for adults. This could be rounded to 20% prevalence or 25% prevalence. There is no obvious method for externally validating one alternative over the other, so it is suggested here that the threshold of 20% rather than 25% is used as the recommended threshold to define a “high” prevalence. It should be recalled that very few countries had obesity prevalence levels as high as 20% only a generation ago. A comparison of the two options showing the distribution of countries across the classifications is shown in Table S2. The proposed criteria for adult obesity are shown in Table 7.

#### 3.3.4 Criteria for prevalence of “at risk of overweight” in children under age 5 years

Children under age 5 years with a weight‐for‐length/height *Z* score between +1.0 and +2.0 are classified as “at risk of overweight” (“at risk”) by the WHO, equivalent to a prevalence between 2.3% at *Z* = +2.0 and 16% at *Z* = +1.0 in the population of children with optimum health. In order to propose prevalence categories for “at‐risk” children, the data for the prevalence of children “at risk” were regressed against the prevalence of children overweight, based on the dataset provided by de Onis et al. in 2010, which provides prevalence figures from 979 surveys in which both “at‐risk” and overweight prevalence values were provided (including repeated surveys over several years).^68^

The surveys were generally in low‐ and lower–middle‐income countries, so the number of countries with higher prevalence levels may have been limited. The results show a good correlation between “at‐risk‐of‐overweight” and overweight prevalence levels (*r* = 0.71, *p* < 0.001) and a regression line close to 0.5 (*y* = 0.52*x* − 0.73) (see Figure S2), indicating that it would be reasonable to use criteria for “at‐risk‐of‐overweight” children at around double those for overweight children in this age group. Using latest available data from each of the 123 different countries gave a distribution in which 23% of countries had a low or very low prevalence of children “at risk of overweight” and 29% of countries had a high or very high prevalence of children “at risk” (see Table S3). Based on the “at risk of overweight” and overweight in combination (i.e., the prevalence of children with weight‐for‐height *Z* scores > +1.0), 27% of countries had a low or very low prevalence, and 31% of the countries had a high or very high prevalence (see Table S3).

#### 3.3.5 Criteria for prevalence of overweight in children 5–19 years

Using the same approach for overweight (without obesity) in older children (defined as having a BMI *Z* score between +1.0 and +2.0 above the median reference population), the prevalence for overweight regressed against that for obesity in this age group, based on 191 countries reported in the WHO Global Health Observatory, shows a strong correlation (*r* = 0.88, *p* < 0.001) and a linear gradient of over 0.9 and an offset of 5 percentage points (*y* = 0.93*x* − 5.20) (see Figure S3). This indicates that the corresponding classifications of prevalence, rounded to convenient levels, can be proposed at 5 percentage points above those for obesity and are shown in Table 7. Of 191 countries, 6% have a “very low” prevalence of overweight children and adolescents, and 16% a “very high” prevalence (see Table S4).

Combining overweight with obesity, the distribution of prevalence levels for all children above a BMI *Z* score of +1.0 shows 7% of countries to have a very low prevalence of overweight including obesity and 14% to have a very high prevalence of overweight including obesity.

The present proposals were compared with those of the World Bank^58^ and the Popkin et al.^59^ paper, which also suggest criteria for prevalence levels for this group of children. The results are shown in Table 5. The World Bank classification scheme appears skewed towards identifying nearly two thirds of countries as having a “very high” prevalence of overweight and obesity. Setting a target for improvement (e.g., bringing the prevalence down to Medium) may be hard to achieve for many of these countries. The Popkin et al.^59^ classification scheme looks less distorted, although nearly 40% of countries are classified as having prevalence levels below “Medium,” which may deter those countries from taking action to prevent child and adolescent overweight from increasing.

**TABLE 5:** Distribution of 191 countries' prevalence levels for overweight and obesity combined for children (aged 5–19 years), comparing proposed classification thresholds, and those of the World Bank Group and Popkin et al.

Proposed classifications	Country distribution	World Bank classifications	Country distribution	Popkin et al. classifications	Country distribution
Very low <10%	7%				
Low 10% to <15%	22%	Low <10%	7%	(Up to 20%)	37%
Medium 15% to <25%	22%	Medium 10% to <15%	22%	Medium >20%	36%
High 25% to <35%	35%	High 15% to <20%	8%	High >30%	21%
Very high ≥35%	14%	Very high ≥20%	63%	Very high >40%	6%
	*N = 191*		*N = 191*		*N = 191*

#### 3.3.6 Criteria for overweight prevalence in adults

Lastly, the categorization of the prevalence of overweight in adults can be proposed. A scatterplot of adult overweight, nonobese prevalence against adult obesity prevalence shows some nonlinearity, which is primarily explained by outliers (mostly in the Pacific Islands) where the mean BMI is above 30 kg/m^2^ (see Figure S4). A linear regression shows a strong correlation (*r* = 0.67, *p* < 0.0001) and a gradient of close to 1 with an offset close to 10 percentage points (*y* = 1.0864*x* − 11.584; see Figure S4). This indicates the rounded thresholds shown in Table 7. [Note that excluding the high BMI outliers and regressing overweight on obesity prevalence only for those countries where obesity prevalence is below 45% gives a correlation of *r* = 0.87 (*n* = 181; *p* < 0.001) and a gradient just over 1 and an offset of 12.4, which would make little difference to the proposed thresholds.]

Table S5 shows the distribution of 191 countries for the prevalence of overweight nonobesity in adults: 18% of countries are classified with a very low or low level of overweight nonobesity in adults, and 58% of countries have a high or very high level of overweight nonobesity in adults. The figures increase marginally to 23% and 59% of countries respectively when overweight and obesity are combined (i.e., prevalence of all adults with BMI > 25 kg/m^2^).

Table 6 shows the distribution for countries using the proposed thresholds for overweight prevalence (including obesity) and compares these with the distributions under the proposed schemes from the World Bank^58^ and Popkin et al.^59^ The latter two classifications schemes are identical, and both would classify two thirds of the world's countries as having a very high prevalence of overweight and obesity combined.

**TABLE 6:** Distribution of 191 countries' prevalence levels for adult overweight and obesity combined, comparing proposed classification thresholds, and those of the World Bank Group and Popkin et al.

Proposed classifications	Country distribution	World Bank classifications	Country distribution	Popkin et al. classifications	Country distribution
Very low <20%	1%				
Low 20% to <30%	22%	Low <20%	1%	(Up to 20%)	1%
Medium 30% to <50%	18%	Medium 20% to <30%	22%	Medium >20%	22%
High 50% to <70%	53%	High 30% to <40%	11%	High >30%	11%
Very high ≥70%	6%	Very high ≥40%	66%	Very high >40%	66%
	*N = 191*		*N = 191*		*N = 191*

## 4 DISCUSSION

The prevalence of undernutrition has been classified since the early 1990s for global monitoring of malnutrition in children under age 5 years. The original threshold level for wasting—the “severity index for malnutrition in emergency situations”—is based on the association of prevalence levels with mortality risk.^56^ Following the publication of the WHO Child Growth Standards in 2006, and the inclusion of child and adolescent overweight and obesity in the 2014 publication *Global Nutrition Targets 2025*, the WHO and UNICEF proposed a set of thresholds of overweight prevalence for the assessment of anthropometric surveys of children under 5 years old.^57^ These joint WHO/UNICEF thresholds are used to define prevalence levels as very low, low, medium, high, and very high.

Prevalence thresholds are used to guide public health intervention policies for communicable diseases as a means of assisting decision‐makers on when it is justified to take population‐level action (e.g., in the case of malaria^69^ or HIV^70^) and have been used for risk assessment purpioses for several years to plan undernutrition interventions. Their use can now be extended for interventions aiming to reduce the risk of overweight and obesity in populations, by alerting health departments and aid agencies to national trends and local needs, by demonstrating progress in policy implementation, and by improving popular understanding of nutrition issues. Prevalence categories can be used to compare countries and their progress towards achieving the World Health Assembly and Sustainable Development Goals for nutrition and can be used by professional organizations to argue the case for strengthening policies to tackle overweight and obesity (an example is the “traffic‐light” coding of childhood obesity levels in the World Obesity Federation report *Atlas of Childhood Obesity*,^60^ and the same organization's categorization of adult obesity levels in their report *Obesity: missing the 2025 global targets* ^61^). In the publications by the World Bank Group^58^ and Popkin et al.,^59^ prevalence categories for child and adult overweight are used in conjunction with measures of undernutrition to demonstrate the dynamics of the double burden of malnutrition, especially in LMICs, and the associated changes in food systems and physical activity that can be identified.

The analyses presented here have led the authors to recommend a set of threshold values for the prevalence of overweight and obesity in populations, presented in Table 7. The thresholds are grounded on the proposals of de Onis et al.^56^ for children under age 5 years used by the WHO and UNICEF and the approach extended by regression analyses to suggest thresholds for categorizing prevalence levels for young children “at risk of overweight” and also to older children and adults, both for obesity prevalence and for overweight prevalence. The proposed classification thresholds are shown in Table 7.

**TABLE 7:** Proposed prevalence classifications for children under age 5 years, aged 5–19 years, and adults, for categories of obesity

Children under 5 years			
	Overweight (WHO > 2sd), obesity (IOTF ≡ BMI > 30 or CDC > 95th)	At risk of overweight only (WHO 1sd to <2sd or IOTF ≡ BMI 25–30 or CDC 85th–95th)	At risk of overweight *and* overweight/obesity (WHO > 1sd or IOTF ≡ BMI > 25 or CDC > 85th)
Very low	<2.5%	<5%	<7.5%
Low	2.5% to <5%	5% to <10%	7.5% to <15%
Medium	5% to <10%	10% to <20%	15% to <30%
High	10% to <15%	20% to <30%	30% to <45%
Very high	≥15%	≥30%	≥45%

Children and adolescents 5–19 years			
	Obesity (WHO > 2sd or IOTF ≡ BMI > 30 or DC > 95th)	Overweight only (WHO 1sd to <2sd or IOTF ≡ BMI 25–30 or CDC 85th–95th)	Overweight including obesity (WHO > 1sd or IOTF ≡ BMI > 25 or CDC ≥ 85th)
Very low	<2.5%	<7.5%	<10%
Low	2.5% to <5%	7.5% to <10%	10% to <15%
Medium	5% to <10%	10% to <15%	15% to <25%
High	10% to <15%	15% to <20%	25% to <35%
Very high	≥15%	≥20%	≥35%

Adults			
	Obesity (BMI ≥ 30)	Overweight only (BMI 25 to <30)	Overweight including obesity (BMI > 25)
Very low	<5%	<15%	<20%
Low	5% to <10%	15% to <20%	20% to <30%
Medium	10% to <20%	20% to <30%	30% to <50%
High	20% to <30%	30% to <40%	50% to <70%
Very high	≥30%	≥40%	≥70%

Abbreviations: ≡ BMI, child's body mass index equivalent to adult's body mass index (IOTF method); CDC, US Centers for Disease Control and Prevention 2000 definitions; IOTF, International Obesity Task Force definitions; WHO, World Health Organization definitions.

The proposed categorizations show many countries in the “high” and “very high” categories: For example, 21% of countries have “high” or “very high” overweight prevalence in children under 5 years, rising to 39% of countries with “high” or “very high” prevalence of obesity in children 5–19 years, and rising further to 55% of countries classified with “high” or “very high” adult obesity prevalence levels.

### 4.1 Limitations

Some care may need to be taken in the use of these thresholds for defining categories of prevalence. Comparisons between countries should be treated carefully, as estimates may be based on different time periods, different subpopulations, different types of survey and measurement methods, or different schemes for defining overweight and obesity. The thresholds proposed here are suggested as covering low‐income, middle‐income, and higher income countries, and across different ethnic and racial groupings, using body mass index (BMI) as the indicator of weight status. Arguments can be made for different thresholds for classifying prevalence in population subgroups or based on different measurement methods for assessing overweight. It should be noted that both the World Bank Group and the Popkin et al. proposals were primarily intended for LMICs, whereas de Onis et al.^56^ did not specify countries where the classification for under‐5s would or would not apply.

Users of prevalence surveys will be aware that populations are constantly in flux and the prevalence levels found in a given survey may lead to a prevalence definition that does not reflect changing circumstances, especially in countries experiencing nutrition transitions or rapid population change. As with many public health measurements, the direction of change over time is as important as the measurement in any one instance.

The methods used in developing the present proposals rely on sets of surveys and estimated prevalence levels published since 2010, and on the use of linear regressions to determine the relation between prevalence levels found in younger age groups and those found in older age groups. Other datasets or alternative means of determining the relationships may have given different results.

The proposed set of thresholds and categories in Table 7 have been applied across the different methods for assessing childhood overweight (developed by WHO, by the US CDC, and by the International Obesity Task Force [IOTF]) without differential analyses. The three approaches do not provide identical estimates of the prevalence of overweight or obesity, and it is possible that the classifications proposed here may not be suitable for surveys using the CDC or IOTF criteria. This remains to be assessed, but for the present, the three approaches have been treated as sufficiently similar to allow the same classification scheme to be applied.

Lastly, as has been noted earlier, there is no external validation for the thresholds and categories proposed here for older children and adults. They are extensions of the approach taken for children under age 5 years, which was linked to the expected prevalence for a healthy population (using *Z* scores) and was internally symmetrical for the proportions of countries in the lowest and highest categories. External validity will depend on the functional value of the thresholds and categories as they are used in practice.

## 5 CONCLUSION

A formal set of criteria for describing and classifying prevalence levels can be of value for policy development and public communication. As the paper by de Onis et al.^56^ concluded, “Harmonized terminology will help avoid confusion and promote appropriate interventions” (p. 175). A review of the use of the descriptor “high” for the prevalence of overweight or obesity found it has been used by researchers somewhat indiscriminately. The present paper proposes a set of threshold values for defining overweight and obesity prevalence in a population as “low,” “medium,” “high,” and so forth based on the approach used by the WHO for children under age 5 years and extended to children aged 5–19 years, and to adults.

As this paper goes to press, these thresholds are being used in a pilot version of the UNICEF–WHO Landscape Analysis tool being applied in several countries in 2020–2021.^5^ The recommended threshold values are also expected to be used in future editions of the World Obesity Federation's *Obesity Atlas* series.

## CONFLICT OF INTEREST

The authors declare no conflict of interests in this work.

## Supporting information


**Table S1.**Distribution of 191 countries according to the prevalence levels for obesity among children aged 5–19 years
**Table S2.**Distribution of 191 countries according to the prevalence levels for obesity among adults: comparison of two ‘medium’ and ‘high’ thresholds
**Table S3.**Distribution of 123 countries according to prevalence of ‘at‐risk of overweight’ and overweight among children under age 5 years.
**Table S4.**Distribution of 191 countries according to prevalence of overweight in children age 5–19 years
**Table S5.**Distribution of 191 countries according to prevalence of overweight among adults
**Figure S1.**Scatterplot of obesity prevalence in children age 5–19 years against obesity prevalence in adults, for 192 countries
**Figure S2.**Scatterplot of ‘at‐risk of overweight’ prevalence against overweight prevalence in children aged under 5 years, for 979 surveys
**Figure S3.**Scatterplot of overweight prevalence against obesity prevalence for children age 5–19 years, for 191 countries
**Figure S4.**Scatterplot of overweight prevalence against obesity prevalence for adults, for 191 countries
