# Supplementary material for: What is a “high” prevalence of obesity? Two rapid reviews and a proposed set of thresholds for classifying prevalence levels
Source: Obes Rev. 2021 Sep 28;23(2):e13363. doi: 10.1111/obr.13363 (PMC9285557; doi:10.1111/obr.13363)
Supplement: Supplementary file 1 — Table S1.Distribution of 191 countries according to the prevalence levels for obesity among children aged 5–19 years Table S2.Distribution of 191 countries according to the prevalence levels for obesity among adults: comparison of two ‘medium’ and ‘high’ thresholds Table S3.Distribution of 123 countries according to prevalence of ‘at‐risk of overweight’ and overweight among children under age 5 years. Table S4.Distribution of 191 countries according to prevalence of overweight in children age 5–19 years Table S5.Distribution of 191 countries according to prevalence of overweight among adults Figure S1.Scatterplot of obesity prevalence in children age 5–19 years against obesity prevalence in adults, for 192 countries Figure S2.Scatterplot of ‘at‐risk of overweight’ prevalence against overweight prevalence in children aged under 5 years, for 979 surveys Figure S3.Scatterplot of overweight prevalence against obesity prevalence for children age 5–19 years, for 191 countries Figure S4.Scatterplot of overweight prevalence against obesity prevalence for adults, for 191 countries [file OBR-23-0-s001.docx]

**Supplementary information to Lobstein T and Jewell J *What is a ‘high’ prevalence of obesity?***

Acknowledgement for datasets: (a) World Health Organization Global Health Observatory (accessed 2021); (b) UNICEF-WHO-UNICEF-World Bank Joint Malnutrition Estimates 2021, and (c) Supplementary data from de Onis M et al *Am J Clin Nutr.* 2010;**92**(5):1257-64.

See main paper for full references.

Acknowledgement for figures: Scatterplots, trend-lines and regression formulae calculated by Microsoft Excel ®

**Supplementary tables**

| Table S.1. | Distribution of 191 countries according to the prevalence levels for obesity among children aged 5-19 years |
| --- | --- |
| Table S.2. | Distribution of 191 countries according to the prevalence levels for obesity among adults: comparison of two ‘medium’ and ‘high’ thresholds |
| Table S.3. | Distribution of 123 countries according to prevalence of ‘at-risk of overweight’ and overweight among children under age 5 years. |
| Table S.4. | Distribution of 191 countries according to prevalence of overweight in children age 5-19 years |
| Table S.5. | Distribution of 191 countries according to prevalence of overweight among adults |

**Supplementary figures**

| Figure S.1. | Scatterplot of obesity prevalence in children age 5-19 years against obesity prevalence in adults, for 192 countries |
| --- | --- |
| Figure S.2. | Scatterplot of ‘at-risk of overweight’ prevalence against overweight prevalence in children aged under 5 years, for 979 surveys |
| Figure S.3. | Scatterplot of overweight prevalence against obesity prevalence for children age 5-19 years, for 191 countries |
| Figure S.4. | Scatterplot of overweight prevalence against obesity prevalence for adults, for 191 countries |

**Table S.1. Distribution of 191 countries according to the prevalence levels for obesity among children aged 5-19 years**

|  | Prevalence thresholds | Countries in each category | |
| --- | --- | --- | --- |
| Proposed rating |  | n |  |
| Very Low | <2.5% | 26 | 14% |
| Low | 2.5% - <5% | 39 | 20% |
| Medium | 5% - <10% | 52 | 27% |
| High | 10% - <15% | 51 | 27% |
| Very High | ≥15% | 23 | 12% |
|  |  | *191* | *100%* |

Source dataset: World Health Organization Global Health Observatory

**Table S.2. Distribution of 191 countries according to the prevalence levels for obesity among adults: comparison of two ‘medium’ and ‘high’ thresholds**

|  | Prevalence thresholds | Countries in each category | | Prevalence thresholds | Countries in each category | |
| --- | --- | --- | --- | --- | --- | --- |
|  |  | n |  |  | n |  |
| Very Low | <5% | 9 | 5% | <5% | 9 | 5% |
| Low | 5% - <10% | 42 | 22% | 5% - <10% | 42 | 22% |
| Medium | 10% - <20% | 35 | 18% | 10% - <25% | 87 | 46% |
| High | 20% - <30% | 81 | 42% | 25% - <30% | 29 | 15% |
| Very High | ≥30% | 24 | 13% | ≥30% | 24 | 13% |
|  |  | *191* | *100%* |  | *191* | *100%* |

Source dataset: World Health Organization Global Health Observatory

**Table S.3. Distribution of 123 countries according to prevalence of ‘at-risk of overweight’ and overweight among children under age 5 years.**

|  | Thresholds for ‘at-risk of overweight’ only (1sd - <2sd) | Countries in each category | | Thresholds for ‘at-risk of overweight’ and overweight (>1sd) | Countries in each category | | Thresholds for overweight only (>2sd) | Countries in each category | |
| --- | --- | --- | --- | --- | --- | --- | --- | --- | --- |
|  |  | n |  |  | n |  |  | n |  |
| Very Low | <5% | 8 | 7% | <7.5% | 8 | 7% | <2.5% | 15 | 12% |
| Low | 5% - <10% | 20 | 16% | 7.5% - <15% | 25 | 20% | 2.5% - <5% | 24 | 20% |
| Medium | 10% - <20% | 59 | 48% | 15% - <30% | 52 | 42% | 5% - <10% | 52 | 42% |
| High | 20% - <30% | 36 | 29% | 30% - <45% | 34 | 28% | 10% - <15% | 18 | 15% |
| Very High | ≥30% | 0 | 0% | ≥45% | 4 | 3% | ≥15% | 14 | 11% |
|  |  | *123* | *100%* |  | *123* | *100%* |  |  |  |

Source dataset: Supplementary tables to de Onis M et al *Am J Clin Nutr.* 2010;**92**(5):1257-64.

**Table S.4. Distribution of 191 countries according to prevalence of overweight in children age 5-19 years**

|  | Thresholds for overweight only (1sd - <2sd) | Countries in each category | | Thresholds for overweight and obesity (>1sd) | Countries in each category | |
| --- | --- | --- | --- | --- | --- | --- |
|  |  | n |  |  | n |  |
| Very Low | <7.5% | 11 | 6% | <10% | 13 | 7% |
| Low | 7.5% - <10% | 41 | 21% | 10% - <15% | 42 | 22% |
| Medium | 10% - <15% | 35 | 18% | 15% - <25% | 42 | 22% |
| High | 15% - <20% | 74 | 39% | 25% - <35% | 67 | 35% |
| Very High | ≥20% | 30 | 16% | ≥35% | 27 | 14% |
|  |  | *191* | *100%* |  | *191* | *100%* |

Source data: World Health Organization Global Health Observatory

**Table S.5. Distribution of 191 countries according to prevalence of overweight among adults**

|  | Thresholds for overweight only  (BMI 25-<30) | Countries in each category | | Thresholds for overweight and obesity  (BMI >25) | Countries in each category | |
| --- | --- | --- | --- | --- | --- | --- |
|  |  | n |  |  | n |  |
| Very Low | <15% | 0 | 0% | <20% | 2 | 1% |
| Low | 15% - <20% | 35 | 18% | 20% - <30% | 42 | 22% |
| Medium | 20% - <30% | 45 | 24% | 30% - <50% | 34 | 18% |
| High | 30% - <40% | 111 | 58% | 50% - <70% | 101 | 53% |
| Very High | ≥40% | 0 | 0% | ≥70% | 12 | 6% |
|  |  | *191* | *100%* |  | *191* | *100%* |

Source data: World Health Organization Global Health Observatory

**Supplementary figures**

**Figure S.1. Scatterplot of obesity prevalence in children age 5-19 years against obesity prevalence in adults, for 192 countries**

Source dataset: World Health Organization Global Health Observatory

**Figure S.2. Scatterplot of ‘at-risk of overweight’ prevalence against overweight prevalence in children aged under 5 years, for 979 surveys**

Source dataset: Supplementary tables to de Onis M et al *Am J Clin Nutr.* 2010;**92**(5):1257-64.

**Figure S.3. Scatterplot of overweight prevalence against obesity prevalence for children age 5-19 years, for 191 countries**

Source dataset: World Health Organization Global Health Observatory

**Figure S.4. Scatterplot of overweight prevalence against obesity prevalence for adults, for 191 countries**

Source dataset: World Health Organization Global Health Observatory
